# Different Income Information as an Indicator for Health Inequality among Japanese Adults

**DOI:** 10.2188/jea.17.93

**Published:** 2007-06-02

**Authors:** Yoshiharu Fukuda, Hiroyuki Nakao, Hirohisa Imai

**Affiliations:** 1 Department of Epidemiology, National Institute of Public Health.

**Keywords:** health inequality, income, self-rated health, smoking, socioeconomic factors

## Abstract

**BACKGROUND:**

There are several alternative indicators of income information, which is a fundamental measure of individual socioeconomic position. In this study, we compared the degrees of associations of four types of income information with health variables among Japanese adults.

**METHODS:**

Using a nationally representative sample of 29,446 men and 32,917 women aged 20 years and over, the associations between four income indicators and health variables were examined using the odds ratio in logistic regression analysis and the concentration index by sex and age group (20-59 years and 60+ years). Income indicators consisted of total household income, equivalent household income, total household expenditure, and equivalent household expenditure. Current smoking and self-rated health statuses were used as health variables.

**RESULTS:**

A low income was associated with a high prevalence of smoking and fair/poor self-rated health, with some differences among sex and age groups and income indicators, but less difference among methods of statistical analyses. Total and equivalent incomes were similarly and more markedly associated with smoking and self-rated health statuses, whereas equivalent expenditure showed the smallest degree of health difference. For the population aged 60+ years, the degree of health differences in smoking was similar between income and expenditure.

**CONCLUSIONS:**

Although the degree of income-related health differences is dependent on health outcome and both sex and age group, this study suggests that either crude or equivalent household income is a useful indicator for health inequality among Japanese adults.

Socioeconomic inequality in health is a major concern in public health.^1^^-^^3^ Many epidemiologic studies have identified inequalities in mortality, morbidity, self-rated health, and health behavior according to individual socioeconomic position.^4^^-^^6^

Measurements of socioeconomic position are fundamental in studies of health inequality because socioeconomic factors feature both as key determinants of health and as critical confounding factors.^7^^-^^9^ Income information is frequently used as a socioeconomic indicator, as well as indicators related to education and occupation.^7^^-^^10^ There are several choices of measurements for income information. In addition to total household income, equivalent income, which is equivalized by household composition (number and/or age of household members), is generally used.^8^^,^^11^^,^^12^ Some previous studies in Japan applied total household income,^13^^-^^17^ whereas others applied equivalent household income.^18^^-^^22^ Information regarding living expenditure or consumption is another alternative representing living standard, and has thus been used in previous studies of health inequality and public health.^23^^-^^26^

Although there is continuous debate about the usefulness of socioeconomic indicators,^27^^-^^29^ no studies have evaluated different income information as an indicator for health inequality in Japan. In this study, we compared the degree of income-related health inequality among several indicators. The inequality was examined using different methods of statistical analyses and according to sex and age group because there are several methods for determining health inequality,^8^^,^^30^ and the degrees of associations between socioeconomic indicators and health variables vary according to both sex and age.^15^^,^^19^^,^^20^^,^^22^ The income indicators were total household income, total household expenditure, and their equivalent values. We used current smoking and self-rated health statuses as health variables, because they were shown to strongly correlate with socioeconomic position in previous studies of the Japanese population.^14^^,^^20^^-^^22^^,^^31^^,^^32^

## METHODS

### Data Source

In this study, we used the 2001 Comprehensive Survey of the Living Conditions of People on Health and Welfare conducted by the Japanese Ministry of Health, Labour and Welfare. The survey comprised interviews with all household members in 5240 area units, sampled at random from all prefectures in Japan, and contained basic household and individual information such as demographics, health, and illness profiles. The total number of households sampled for basic information was 247,195, and 30,387 of these households were additionally interviewed about their income and savings.^33^ Microdata files from the survey were used with official permission from the Ministry of Internal Affairs and Communication. We restricted our analyses to data of 29,227 households and their household members (29,446 men and 32,917 women) aged 20+ years whose income and health variables shown below were available.

### Income Indicators

All households in the basic survey were interviewed regarding their monthly household expenditure. In the income and savings survey, the interviews included questions regarding annual household income before tax, including benefits and inheritance. In addition to the total household income and expenditure, we used equivalent income and expenditure to take into account differences in household size and composition. The total household income and total household expenditure were divided by the household’s equivalent adult size, using the modified Organization for Economic Cooperation and Development (OECD) equivalent scale. This scale gave a weight to the first adult, 0.5 to the second and each subsequent person aged 14 years and over, and 0.3 to each child aged less than 14 years in the household.^11^ The quintiles or dectiles of the four income indicators were used in the following analyses.

### Health Variables

Smoking status was surveyed using the following four categories: (1) I do not smoke; (2) I smoke every day; (3) I smoke occasionally; and (4) I have stopped smoking for more than one month. Subjects whose responses were (2) and (3) were categorized as current smokers, and (1) and (4) as noncurrent smokers. Self-rated health was determined as excellent, very good, good, fair, or poor. We formed a dichotomous variable of excellent/very good/good and fair/poor.

### Analysis

We applied three approaches to determine the associations between income indicators and health variables.

First, the odds ratio (OR) with 95% confidence interval of current smoking and fair/poor self-rated health for the lowest income quintile compared with the highest income quintile was calculated using logistic regression analysis. Second, the ORs of current smoking and fair/poor self-rated health across income quintiles were calculated using logistic regression analysis with income quintiles as ordinal variables: the quintiles were assigned 0.1 (highest income), 0.3, 0.5, 0.7, and 0.9 (lowest income). This method was based on a linear trend relationship and the principle of the relative index of inequality, which can be interpreted as the odds ratio of smoking or fair/poor self-rated health for the bottom of the income hierarchy as compared with the top.^8^^,^^34^ A multilevel model with individuals (level 1) nested within 47 prefectures (level 2) was used to take into account regional variation in income and health variables.^18^^,^^19^ The iterative generalized least-square (IGLS) was fitted for estimation of OR with 95% confidence interval using MLwiN^®^ 2.0.^35^ Adjustment for age as a potential confounding factor was made in the above analyses.

Third, the concentration index was used.^30^^,^^36^ It was defined with reference to the concentration curve, with the cumulative percentage of the sample ranked by income beginning with the poorest on the x-axis, and the cumulative percentage of the health variable corresponding to each cumulative percentage of the distribution of the income indicator on the y-axis. The concentration index was calculated as twice the area between the concentration curve and the line of equality (the 45° line running from the bottom left corner to the top right), ranging from −1.0 to 1.0. The index is zero in the absence of income-related inequality. We calculated the index using dectile categories of the income indicator.^36^

The analyses were conducted separately according to sex and age group because the relationship between socioeconomic indicators and health variables depends on sex and age.^15^^,^^19^^,^^20^^,^^22^ The subjects were divided into those aged 20-59 years and those aged 60+ years, considering differences in social conditions such as employment status. Categorization of quintiles and dectiles was also conducted separately according to sex and age group.

## RESULTS

Table 1 shows the proportions of current smokers and of the sample population with fair/poor self-rated health. Fifty percent of men and 12.4% of women were smokers, and 15.0% of men and 17.7% of women rated their health status as fair or poor. The younger group showed a higher proportion of smokers and a lower proportion with fair/poor self-rated health for both men and women.

**Table 1.** Proportions of current smokers and sample population with fair/poor self-rated health.

Age (year)	n	Current smoker (%)	Fair/poor self- rated health (%)
Male			
Total	29,446	14,699 (49.9%)	4407 (15.0%)
20-59	20,102	11,518 (57.3%)	2183 (10.9%)
60+	9344	3181 (34.0%)	2224 (23.8%)

Female			
Total	32,917	4083 (12.4%)	5828 (17.7%)
20-59	21,108	3388 (16.1%)	2633 (12.5%)
60+	11,809	695 (5.9%)	3195 (27.1%)

Distributions of the four income indicators are shown in Table 2, showing percentiles and the Gini coefficient for the total 29,227 households included in this study. On the basis of the Gini coefficient, the total income showed the largest variation, followed by the equivalent income and the equivalent expenditure.

**Table 2.** Distribution of four income indicators (10,000 yen, 29,227 households).

Income-related indicator		Percentile					Gini coefficient

		5	25	50	75	95	
Household income (annual)	Total	90	267	486	800	1452	0.40
	Equivalent	66.7	166.0	262.7	395.2	720.0	0.36

Household expenditure (monthly)	Total	8	15	23	30	54	0.36
	Equivalent	5.0	9.3	1 2.5	16.7	30.0	0.34

Table 3 shows the results of logistic regression analysis for the lowest-income quintile compared with the highest-income quintile according to different income indicators. The ORs were adjusted for age, which was significantly (p<0.001) associated with the smoking negatively and with fair/poor self-rated health positively. Household income showed the strongest association with the female smoking status, followed by the male self-rated health status. The group aged 20-59 years showed a higher OR than the group aged 60+ years, except for the male self-rated health status. The ORs of both health variables for the total and equivalent incomes were similar regardless of sex and age group. Although the OR of the smoking status for the total expenditure in the group aged 60+ years was similar to those for the total and equivalent incomes, the association of the smoking status with expenditure was generally weaker than that with income. The household expenditure for the female self-rated health status did not show a significant positive association.

**Table 3.** Odds ratios* (95% confidence intervals) of smoking and fair/poor self-rated health for lowest quintile compared with highest quintile, according to four income indicators.

Health variable	Sex	Age (year)	Household income		Household expenditure	
	
			Total	Equivalent	Total	Equivalent
Smoking	Male	20-59	1.31 (1.20-1.43)	1.36 (1.24-1.49)	1.01 (0.92-1.10)	1.02 (0.93-1.11)
		60+	1.24 (1.08-1.43)	1.26 (1.10-1.45)	1.33 (1.16-1.52)	1.36 (1.18-1.56)

	Female	20-59	2.84 (2.50-3.22)	2.29 (2.02-2.60)	1.63 (1.45-1.83)	1.15 (1.02-1.30)
		60+	1.98 (1.56-2.53)	1.88 (1.48-2.40)	1.71 (1.34-2.18)	1.25 (0.97-1.61)

Fair/poor self-rated health	Male	20-59	1.56 (1.35-1.79)	1.45 (1.26-1.66)	1.20 (1.05-1.37)	1.09 (0.95-1.25)
		60+	1.76 (1.51-2.05)	2.02 (1.72-2.37)	1.12 (0.97-1.30)	1.12 (0.96-1.31)

	Female	20-59	1.48 (1.30-1.69)	1.30 (1.14-1.48)	1.00 (0.88-1.13)	0.84 (0.74-0.96)
		60+	1.34 (1.18-1.52)	1.33 (1.17-1.52)	0.99 (0.87-1.12)	0.93 (0.82-1.07)

* : Adjusted for age

Table 4 shows the results of logistic regression analysis according to the ordinal variables of income indicators. The results of this analysis were similar to those shown in Table 3. The association of two health variables with household income was more pronounced than that with household expenditure, except for the smoking status in men aged 60+ years. The total and equivalent incomes showed similar positive associations, whereas the equivalent expenditure did not show a significant positive association with the smoking status or self-rated health status for a certain sex and some sex and age groups.

**Table 4.** Odds ratios* (95% confidence intervals) of smoking and fair/poor self-rated health according to ordinal variables of four income indicators.

Health variable	Sex	Age (year)	Household income		Household expenditure	
	
			Total	Equivalent	Total	Equivalent
Smoking	Male	20-59	1.44 (1.30-1.60)	1.49 (1.35-1.66)	1.02 (0.92-1.12)	1.05 (0.95-1.16)
		60+	1.25 (1.07-1.47)	1.34 (1.15-1.56)	1.36 (1.17-1.58)	1.42 (1.22-1.66)

	Female	20-59	3.82 (3.32-4.39)	2.96 (2.58-3.40)	1.72 (1.51-1.97)	0.99 (0.86-1.13)
		60+	2.63 (1.97-3.51)	2.31 (1.74-3.07)	2.01 (1.52-2.67)	1.33 (1.01-1.77)

Fair/poor self-rated health	Male	20-59	1.72 (1.47-2.02)	1.53 (1.30-1.79)	1.24 (1.07-1.45)	1.09 (0.93-1.27)
		60+	2.00 (1.68-2.38)	2.35 (1.98-2.80)	1.17 (0.99-1.39)	1.18 (1.00-1.40)

	Female	20-59	1.53 (1.32-1.77)	1.38 (1.19-1.60)	0.99 (0.85-1.14)	0.82 (0.71-0.95)
		60+	1.36 (1.17-1.57)	1.41 (1.22-1.64)	1.01 (0.87-1.17)	0.95 (0.82-1.11)

* : Adjusted for age

The concentration index of current smoking and fair/poor self-rated health according to different income indicators are shown in Table 5. A negative index indicates that current smoking or fair/poor self-rated health was likely to be concentrated in lower income categories. For male smokers, the absolute concentration indices were low, indicating modest inequality among male smokers. High absolute concentration indices were found among female smokers, particularly for the total income, followed by the equivalent income. For the self-rated health status, the concentration indices of the total and equivalent incomes were similar and markedly higher than those of the total and equivalent expenditure. For women, the total and equivalent expenditures showed inverse associations (positive concentration index) with health variables in some cases.

**Table 5.** Concentration index of smoking and fair/poor self-rated health according to four income indicators.

Health variable	Sex	Age (year)	Household income		Household expenditure	
	
			Total	Equivalent	Total	Equivalent
Smoking	Male	20-59	-0.030	-0.033	-0.006	-0.011
		60+	-0.013	-0.017	-0.021	-0.022

	Female	20-59	-0.181	-0.150	-0.083	-0.006
		60+	-0.136	-0.108	-0.084	-0.003

Fair/poor self-rated health	Male	20-59	-0.069	-0.047	-0.019	0.004
		60+	-0.094	-0.117	-0.033	-0.040

	Female	20-59	-0.052	-0.034	0.009	0.044
		60+	-0.031	-0.048	-0.003	-0.015

## DISCUSSION

In this study, we compared the degree of associations of different income indicators with smoking status and self-rated health status in the Japanese population. Although there were slightly different patterns according to sex, age group, and health variables, the indicators of household income, either crude or equivalent, generally showed stronger associations than those of household expenditure. The equivalent household expenditure showed the weakest associations with the smoking and self-rated health statuses. The same findings were obtained regardless of the method of statistical analysis.

In addition to educational and occupational information, income is commonly used as a measurement of individual socioeconomic position in health inequality studies.^8^^-^^10^ However, there are a few disadvantages in using income information. First, personal income is a delicate issue; thus, people may be reluctant to provide their actual income information.^7^^,^^8^^,^^37^ Second, additional information on household members is required to obtain equivalent income for comparability across households. Compared with income, household expenditure is easier to acquire and provides a better representation of current living standards and material wealth than household income.^23^ Therefore, household expenditure has been used in a number of national surveys, including the basic survey used in this study,^33^ in addition to individual epidemiologic studies.^24^^-^^26^

The results of this study indicated that household income, either total or equivalized by the composition of household members, is more sensitive to health inequality than household expenditure. Regarding household expenditure, the total expenditure showed weak but significant associations with both smoking and self-rated health statuses, whereas the equivalent expenditure did not show clear associations. Thus, when household expenditure is used as income information, the total, not equivalent, expenditure appears to be suitable. The use of less sensitive indicators such as equivalent expenditure will underestimate health inequality.

The reason why the association of health variables with household income was stronger than that with household expenditure was not determined in this study. It is possible that household income reflects other socioeconomic factors, including educational attainment, occupational status, and socioeconomic position, throughout the course of a person’s life more closely than household expenditure. The more pronounced health status differences according to household income are probably due to the effect of other socioeconomic factors as reflected by household income.

The strengths of this study include the use of both household income and expenditure of a large study sample from across the country. In addition, we applied two different health variables and different established methods of statistical analyses to identify health inequality.^8^^,^^30^^,^^34^ Similar patterns of differences according to income information were found regardless of the method used for statistical analysis.

This study has several limitations. First, income information was self-reported. Information about household income was obtained by interview using detailed items; thus, it might be more reliable than expenditure information. This fact may partly explain the stronger association between income and health variables.

Second, only two health variables were used, namely, smoking and self-rated health statuses, which are the most common variables strongly associated with socioeconomic indicators.^13^^-^^15^^,^^19^^-^^22^^,^^31^^,^^32^ It is possible that other health variables show degrees of association with income indicators different from those in this study.

Third, it is not clear whether the results of this study can be generalized to other populations, particularly those in other countries. Although health inequality has been demonstrated in most countries, the pattern of inequality differs between countries.^34^^,^^38^^-^^41^ Differences in the meaning of income and expenditure, and the structure and size of the household between countries possibly result in variations in income-related health inequality.

Finally, important confounding factors might not be considered; thus, we were not able to identify independent effects of income. Indicators of education and occupation are generally associated with smoking and self-rated health statuses.^13^^,^^14^^,^^22^^,^^31^^,^^39^^,^^40^ Social conditions such as marital status, living arrangement, and social support might be critical for both smoking and self-rated health statuses.^20^^,^^21^^,^^42^^,^^43^ On the basis of our present findings, the independent and interactive associations of income and other socioeconomic indicators with health variables need to be examined in future studies.

In conclusion, results of this study suggest that income-related differences in smoking and self-rated health statuses can be detected using household income, either the total or equivalent, more sensitively than using household expenditure. The notably good health status of the Japanese population may be attributable to a small degree of socioeconomic disparities.^44^^,^^45^ Nevertheless, recent studies demonstrated substantial differences in health status according to the individual socioeconomic position in Japan.^13^^-^^16^^,^^19^^-^^22^^,^^31^^,^^32^^,^^46^^,^^47^ The findings of this study will contribute to future analysis and debate on health inequality among the Japanese population.
